# eARDS: A multi-center validation of an interpretable machine learning algorithm of early onset Acute Respiratory Distress Syndrome (ARDS) among critically ill adults with COVID-19

**DOI:** 10.1371/journal.pone.0257056

**Published:** 2021-09-24

**Authors:** Lakshya Singhal, Yash Garg, Philip Yang, Azade Tabaie, A. Ian Wong, Akram Mohammed, Lokesh Chinthala, Dipen Kadaria, Amik Sodhi, Andre L. Holder, Annette Esper, James M. Blum, Robert L. Davis, Gari D. Clifford, Greg S. Martin, Rishikesan Kamaleswaran

**Affiliations:** 1 Department of Biomedical Informatics, Emory University School of Medicine, Atlanta, Georgia, United States of America; 2 Division of Pulmonary, Critical Care, Allergy, and Sleep Medicine, Emory University School of Medicine, Atlanta, Georgia, United States of America; 3 Department of Pediatrics, University of Tennessee Health Science Center, Memphis, Tennessee, United States of America; 4 Department of Medicine, University of Tennessee Health Science Center, Memphis, Tennessee, United States of America; 5 Department of Anaesthesia, Emory University School of Medicine, Atlanta, Georgia, United States of America; 6 Department of Biomedical Engineering, Georgia Institute of Technology, Atlanta, Georgia, United States of America; Ohio State University Wexner Medical Center Department of Surgery, UNITED STATES

## Abstract

We present an interpretable machine learning algorithm called ‘eARDS’ for predicting ARDS in an ICU population comprising COVID-19 patients, up to 12-hours before satisfying the Berlin clinical criteria. The analysis was conducted on data collected from the Intensive care units (ICU) at Emory Healthcare, Atlanta, GA and University of Tennessee Health Science Center, Memphis, TN and the Cerner^®^ Health Facts Deidentified Database, a multi-site COVID-19 EMR database. The participants in the analysis consisted of adults over 18 years of age. Clinical data from 35,804 patients who developed ARDS and controls were used to generate predictive models that identify risk for ARDS onset up to 12-hours before satisfying the Berlin criteria. We identified salient features from the electronic medical record that predicted respiratory failure among this population. The machine learning algorithm which provided the best performance exhibited AUROC of 0.89 (95% CI = 0.88–0.90), sensitivity of 0.77 (95% CI = 0.75–0.78), specificity 0.85 (95% CI = 085–0.86). Validation performance across two separate health systems (comprising 899 COVID-19 patients) exhibited AUROC of 0.82 (0.81–0.83) and 0.89 (0.87, 0.90). Important features for prediction of ARDS included minimum oxygen saturation (SpO_2_), standard deviation of the systolic blood pressure (SBP), O_2_ flow, and maximum respiratory rate over an observational window of 16-hours. Analyzing the performance of the model across various cohorts indicates that the model performed best among a younger age group (18–40) (AUROC = 0.93 [0.92–0.94]), compared to an older age group (80+) (AUROC = 0.81 [0.81–0.82]). The model performance was comparable on both male and female groups, but performed significantly better on the severe ARDS group compared to the mild and moderate groups. The eARDS system demonstrated robust performance for predicting COVID19 patients who developed ARDS at least 12-hours before the Berlin clinical criteria, across two independent health systems.

## Introduction

The novel coronavirus-2019 disease (COVID-19) pandemic has led to a disruptive global health crisis with significant morbidity and mortality. It has placed a significant burden on the healthcare system, with about 15–29% of COVID-19 cases requiring hospitalization, and about 17–35% of inpatients requiring critical care [1–4]. The morbidity and mortality among the critically ill patients with COVID-19 is particularly high, especially related to respiratory failure and acute respiratory distress syndrome (ARDS). Some prior studies have suggested that the risk of ARDS in mechanically ventilated patients with COVID-19 ranges 40–100% [5–7], and the mortality in those requiring mechanical ventilation is reported to be as high as 50–97%—higher than the mortality rates from other causes of ARDS, including H1N1 influenza [5, 6, 8].

Although ARDS secondary to COVID-19 may satisfy the Berlin definition of ARDS, some features that appear distinct from “classic” ARDS have also been suggested [9, 10]. Such differences include preservation of the respiratory system compliance despite severe hypoxemia in some patients [9, 11], as well as relatively delayed timing of onset compared to the 7-day period included in the Berlin definition [12]. Based on the differences in respiratory system compliance and hypothesized mechanisms of hypoxemia, some studies have proposed subphenotypes of COVID-19 induced ARDS that may behave differently from “classic” ARDS, such as the high- and low-elastance phenotypes [9, 13]. The differences between the subphenotypes were also corroborated by a study of computed tomographic examinations of the lungs in COVID-19 and non-COVID-19 ARDS patients [14]. Although the validity and the clinical significance of these differences between COVID-19 ARDS and “classic” ARDS is uncertain and debatable [15], they nonetheless highlight the heterogeneity in COVID-19 induced respiratory failure and ARDS. The heterogeneity and potentially distinct features of COVID-19 ARDS, combined with the aforementioned high mortality, present unique challenges for its diagnosis, risk-stratification, and management. These new challenges are also applicable for predictive modeling in ARDS. While several prior studies have utilized machine learning models to identify and/or predict general ARDS [16–19], these models have not been trained or validated on populations containing patients with COVID-19.

The high incidence of and mortality from ARDS in COVID-19 highlights an important need for early prediction and recognition of ARDS in this population. Machine learning models for predicting ARDS that are validated in COVID-19 patients have the potential to improve early identification of patients who are at high risk of disease progression and promote timely implementation of indicated treatments. The objective of this study was to address this need by developing and validating a machine learning model for early prediction of ARDS development and its severity in COVID-19 patients before they satisfy the clinical definition of ARDS. In this paper, we introduce a machine learning model called *eARDS*, which predicts the onset of ARDS in critically ill COVID-19 patients up to 48 hours before meeting the Berlin definition.

## Materials and methods

This study was approved by the Institutional Review Board at Emory University, Atlanta GA (#IRB00033069), and The University of Tennessee Health Science Center, Memphis TN (#20-07294-XP).

### Description of the datasets

The model was derived from the Cerner Real-World Data, consisting of de-identified information from hospitals within the Cerner environment, and was evaluated on patients who developed ARDS during hospitalization at the Emory Healthcare and the UTHSC-Methodist LeBonheur Healthcare systems for patients with a positive SARS-CoV-2 result by qRT-PCR. The eARDS model was trained using the de-identified Cerner Real-World Data^™^ (CRD), which consists of both COVID19 and non-COVID19 patients from hospitals across various geographic regions and demographics. Data was captured between January till April 2020, and did not include any patients from the Emory healthcare or UTHSC-MLH system.

For validation of the eARDS, we derived data from 767 COVID-19 patients admitted across 4 hospitals within the Emory Healthcare system, Atlanta, GA, and 132 COVID-19 patients admitted across 5 hospitals within the Methodist LeBonheur Healthcare (MLH) system, Memphis TN. Demographic information, medical comorbidities, vital signs, laboratory data, and other clinical information abstracted from the electronic health records (EHR) (S1 Table) were selected from admission till the onset of ARDS from the ICU. These variables were selected based on the literature pertaining to the prediction of ARDS. We extracted data for patients from February to June 1, 2020.

### Selection criteria

All patients above 18 years of age who were admitted to the ICU diagnosed with SARS-CoV-2 with at least 48 hours of data were included in the study. Where we observed multiple encounters for the same patient, we treated each encounter as independent if the admissions were at least 30 days apart, and used the first admission. We used Current Procedural Terminology (CPT) and International Classification of Diseases (ICD-10) to identify mechanical ventilation and oxygen therapy. The onset time of ARDS is measured using the Berlin criteria [20], in which we identify t_Onset_ as when the patient requires positive end-expiratory pressure (PEEP) of at least 5 cmH_2_O and a ratio of arterial partial pressure oxygen to fraction of inspired oxygen (P/F) ratio < = 300, within 1 week of initial oxygen support. We further exclude patients based on the percentage missing data i.e., if the percentage *null* value is greater than 90%. We further segregate ARDS patients by severity, using the worst P/F during an encounter, specifically the severity classes were: mild (P/F ratio > = 200 and <300), moderate (P/F ratio > = 100 and <200), and severe (P/F ratio < 100).

### Missing data and class imbalanced

All the missing values of laboratory and vital sign measurements were filled using last-one carry forward imputation and remaining missing values were imputed by the global median of the associated variable in the training dataset (CRD dataset) and validation dataset (Emory and UTHSC-MLH dataset). Separate binary variables were generated to indicate a positive binary value during their oxygen therapy, mechanical ventilation, vasoactive status and for their comorbidities. Class imbalance was addressed through balanced micro batching, in which we balance ARDS patients with an equal random set of Non-ARDS patients.

### Data preprocessing

The data of each patient was segmented and resampled at an evenly sampled 2-hour interval. Their median value replaced the multiple measures available for a single variable within the 2-hour interval. Leaving an interval of 12-hours before ARDS onset as the *prediction window*, we further segment data in 16-hour *observational windows* to extract statistical features. From continuous variables, we extracted features including, minimum, maximum, standard deviation, median, and skewness. For categorical variables, such as the presence of therapies or medication, we generated a binary flag to indicate the presence of the variable at the appropriate time intervals. In this manner, we obtained 148 statistical features that are provided as inputs to our model.

### Model development and evaluation

We developed our machine learning models on Python using the Scikit-Learn [21] and the XGBoost package [22]. Data management was performed using the Pandas library [23]. During the course of the machine learning pipeline, we evaluated a number of machine learning methods including, Neural Networks [24], Support Vector Machines [25], Random Forests [26], Logistic Regression [27], and eXtreme Gradient Boosting (XGBoost) [28]. We then selected the XGBoost model due to the robust and superior performance across the internal and external validations. In the derivation of eARDS was performed in two steps, first, a model was trained on 80% of the CRD database (training set) which consisted of ARDS in patients positive for SARS-CoV-2. The remaining 20% were preserved as a hold-out dataset, this process was repeated with a random selection of patients and repeated 10 times with replacement to generate average training performance. Prior to training a model, we used a subset of the training data (30%) for hyperparameter selection using Bayesian optimization.

We then validated the model on retrospective data collected on COVID-19 patients from Emory and UTHSC-MLH datasets. We selected a random 80% of the dataset and repeatedly sampled from this dataset 10 times with replacement to generate confidence intervals of the performance statistics.

### Feature importance and model interpretability

A popular recent method for explaining machine learning is by the use of SHapley Additive exPlanations (SHAP) [29], which uses optimal credit allocations among entities to derive their contributions, a game theory centric method for feature importance at the prediction level. We used SHAP to extract prediction level explanations along with mean SHAP values generated across predictions to develop interpretations of important predictors.

## Results

### Clinical characteristics

Table 1 shows the clinical characteristics of the study population. In the training dataset, 35,804 patients were available, of which 14,097 met inclusion criteria. 1,890 patients (13.4%) met ARDS criteria, and of that 964 were positive for SARS-CoV-2. Among the 12,207 patients who did not meet ARDS criteria, 4,712 were positive for SARS-CoV-2. The median age of ARDS patients was 66 [54, 77] while for Non-ARDS, it was 60 [44, 73], the number of males (%) for ARDS is 1049 (56%) and for Non-ARDS is 5,966 (49%). Statistically significant differences (P < 0.05) were found among gender, race, and ethnicity in the training dataset.

**Table 1:** Characteristics of patients in the datasets.

	Emory		UTHSC-MLH		Cerner Real-World Data	
	ARDS	NON-ARDS	ARDS	NON-ARDS	ARDS	NON-ARDS
**Patients, n**	145	466	17	60	1890	12207
**COVID-19, n (column %)**	145 (100)	466 (100)	17 (100)	60 (100)	964 (51%)	4712 (39%)
**Age, mean [IQR]**	^+^67 [58, 77]	^+^61 [50, 73]	68 [59, 80]	62 [52, 73]	66 [54, 77]	60 [44, 73]
**Age Groups, n (column %)**						
18 yrs. to 40 yrs.	^#^10 (7%)	56 (12%)	0 (0%)	1 (2%)	^#^148 (8%)	2342 (19%)
41 yrs. - 60 yrs.	30 (21%)	155 (33%)	6 (35%)	26 (43%)	536 (28%)	3754 (31%)
61 yrs. - 80 yrs.	75 (51%)	194 (42%)	6 (35%)	24 (40%)	828 (44%)	4188 (34%)
81+ yrs.	30 (21%)	61 (13%)	5 (30%)	9 (15%)	378 (20%)	1923 (16%)
**Male, number (column %)**	82 (57%)	223 (48%)	8(47%)	30(50%)	^#^1,049 (56%)	5,966 (49%)
**First Day Apache II Score** * **, mean [IQR]**	19 [13, 26]	15 [12, 20]	19 [13, 26]	12 [8, 15]	19 [11, 34]	16 [8, 28]
**Mechanical Ventilation, n (column %)**	^#^94 (65%)	34 (7%)	^#^7 (41%)	5 (8%)	^#^889 (47%)	755 (6%)
**P/F Ratio, mean (IQR)**	^+^215 [158, 328]	341 [207, 341]	^+^200 [145, 340]	340 [272, 340]	^+^161 [107, 231]	312 [170, 393]
**Race, n (column %)**						
African American	94 (65%)	313 (68%)	15 (88%)	49 (82%)	^#^518 (27%)	3,100 (25%)
Caucasian	40 (28%)	92 (20%)	0 (0%)	7 (12%)	916 (49%)	6,407 (53%)
American Indian	0 (0%)	1 (0%)	0 (0%)	0 (0%)	31 (2%)	226 (2%)
Asian	2 (1%)	9 (2%)	0 (0%)	1 (2%)	59 (3%)	356 (3%)
Mixed Race	1 (0%)	0 (0%)	1 (6%)	1 (2%)	1 (0%)	0 (0%)
Unknown Race	8 (6%)	51 (10%)	1 (6%)	2 (3%)	365 (19%)	2117 (17%)
**Ethnicity, n (column %)**						
Not Hispanic or Latino	^#^134 (92%)	386 (83%)	16 (94%)	59 (98%)	^#^1,323 (70%)	8,461 (69%)
Hispanic or Latino	4 (3%)	22 (5%)	1 (6%)	1 (2%)	330 (17%)	2,518 (21%)
Unknown	7 (5%)	58 (12%)	-	-	237 (13%)	1228 (10%)

*Excludes Chronic Health Points due to lack of data availability.

^+^statistical significance by Wilcoxon Rank-Sum test (P < 0.05).

^#^statistical significance by Chi-square test (P < 0.05).

In the validation datasets, a total of 767 COVID-19 patients were available from Emory Healthcare, and 611 met the inclusion criteria and were included in the analysis. Of these, 145 were ARDS patients and 466 were Non-ARDS patients. There were 132 COVID-19 patients available from the UTHSC-MLH dataset, and 77 patients met inclusion criteria and were included in the analysis. Of these, 17 patients met the ARDS criteria. The average age among patients across both datasets was similar, with the UTHSC-MLH patients being a year older on average in both groups. Comparing ARDS and non-ARDS patients did not show statistical significance differences (P < 0.05) in age, gender, race, or ethnicity. Among the Emory dataset, however, we observed statistically significant differences in age and ethnicity (P < 0.05).

### Data preprocessing

There were a number of challenges in the preprocessing of the CRD dataset. The most prominent of which were discrepancies among units in similar measures, for instance, FiO_2_ values were present in both fractional and percentage forms at various periods, which required standardization, and temperature appeared in both Celsius and Fahrenheit. We further observed variable contamination, for e.g. heart rate with a unit of beats/min appeared labeled as ‘mean arterial pressure’, which required explicit unit validation. Some measures were erroneously high in this dataset which were categorized as values greater than 99 percentile value for that variable, these were imputed with the global median.

**Fig 1:**
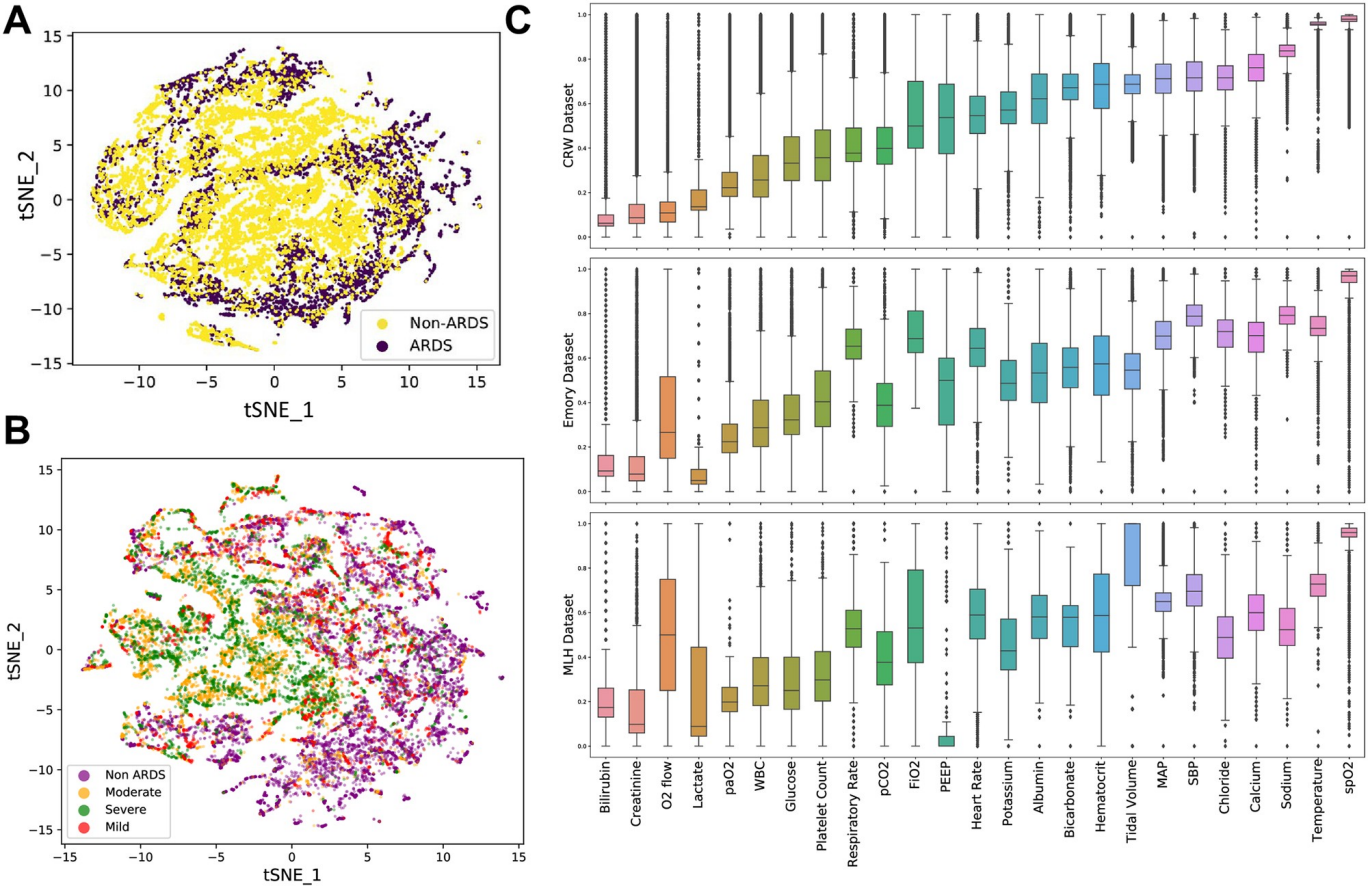
Characteristic analysis of the data. A. Illustrates a t-SNE plot of ARDS vs non-ARDS comparing across all predictors, two localised clusters emerge consisting of ARDS (yellow) patients; B. t-SNE plot of ARDS Severity, classified as mild, moderate and severe, significant convergence can be observed among the moderate and severe groups with non-ARDS and mild groups similarly forming a separate cluster. C. A box-plot of data density is illustrated, with each of the variables compared across the three data sources. Statistical significance is observed in all variables except PaO_2_ and Chloride.

From validation datasets, we observed some challenges in preprocessing the UTHSC-MLH dataset, particularly as some structured data fields contained text data and/or comments relevant for clinical interpretations. Negative values were observed in fields, which were flagged as erroneous by clinical experts. In the Emory dataset, we observed inconsistencies in some values being present in fractional and percentage forms, which required standardization. Fig 1(A) illustrates the tSNE plot of ARDS vs Non-ARDS patients using the preprocessed data. Significant clustering around the center was observed for the ARDS patients (in yellow), while Non-ARDS (in purple) are clustered around the periphery. Fig 1(B) represents the tSNE plot between Non-ARDS and ARDS severity. A summary of the normalized preprocessed data across each of the three datasets are illustrated in box-plots in Fig 1(C), statistical differences were observed in all variables between the three groups, except for PaO_2_ and Chloride. S1 Fig illustrates a box-plot figure of missingness, i.e. the percentage of missing values that existed for each variable across the different datasets.

### Training and validation model performance

**Fig 2:**
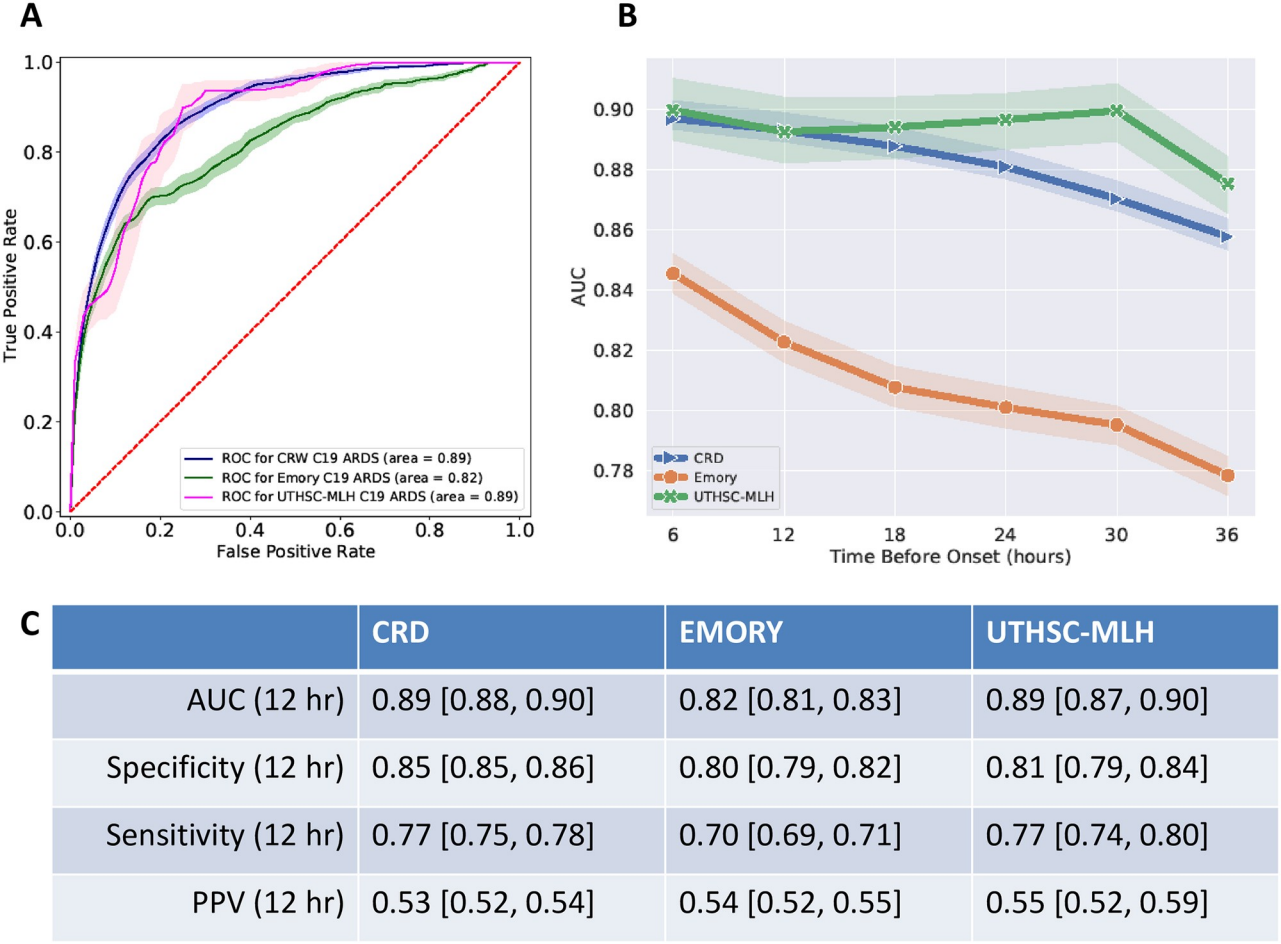
Model performance and feature importance. A. The AUROC performance of the eARDS model over hold-out and validation datasets, 95% CI, are illustrated as shaded bands for each model. B. Temporal performance of the model over different prediction horizons ranging from t_onset_ to 36 hours before, MLH is shown to have the highest performance over the time periods, while Emory data has the lowest AUC consistently. C. Depicts the performance measures of the algorithm across the three datasets at 12-hours before ARDS onset.

Performance measures derived from evaluating eARDS on the 20% CRD hold-out, and the validation results (UTHSC-MLH and Emory) are summarized in Table 2. As illustrated in Fig 2(A), at 12-hours before ARDS onset, the training model achieved an AUC [95% CI] of 0.89 [0.88, 0.90] averaged over 10 iterations of bootstrap. The sensitivity, specificity and positive predictive value (PPV), of the hold-out CRD dataset were 0.77 [0.75, 0.78], 0.85 [0.85, 0.86] and 0.53 [0.52, 0.54], respectively.

**Table 2:** Analysis of model performance over 6-hour and 12-hour prediction horizon.

**COVID-19 Positives (6 hours)**			
Dataset	CRW	Emory	MLH
AUC	0.90 [0.889, 0.90]	0.85 [0.85, 0.86]	0.88 [0.86, 0.90]
Specificity	0.86 [0.86, 0.86]	0.81 [0.80,0.82]	0.82 [0.80, 0.84]
Sensitivity	0.77 [0.76,0.78]	0.74 [0.73, 0.76]	0.77 [0.74, 0.78]
PPV	0.54 [0.53, 0.54]	0.55 [0.54, 0.59]	0.56 [0.53, 0.59]
**COVID-19 Positives (12 Hours)**			
Dataset	CRW	Emory	MLH
AUC	0.89 [0.88, 0.90]	0.82 [0.81, 0.83]	0.89 [0.87, 0.90]
Specificity	0.85 [0.85 0.86]	0.80 [0.79, 0.82]	0.81 [0.79, 0.84]
Sensitivity	0.77 [0.75, 0.78]	0.70 [0.69, 0.71]	0.77 [0.74, 0.80]
PPV	0.53 [0.52, 0.54]	0.54 [0.52, 0.55]	0.55 [0.52, 0.59]
**All-cause ARDS (12 Hours)**			
Dataset	CRW	Emory	MLH
AUC	0.86 [0.85, 0.86]	0.80 [0.79, 0.81]	0.86 [0.84, 0.87]
Specificity	0.79 [0.79, 0.80]	0.77 [0.76, 0.78]	0.79 [0.76, 0.81]
Sensitivity	0.77 [0.76, 0.77]	0.70 [0.69, 0.71]	0.75 [0.72, 0.78]
PPV	0.45 [0.44, 0.45]	0.49 [0.48, 0.51]	0.51 [0.48, 0.55]

For the same time period, among the validation datasets, we observed an AUC for Emory and UTHSC-MLH of 0.82 [0.81, 0.83] and 0.89 [0.87, 0.90], respectively, the sensitivity, specificity, and PPV are included in Table 2. At 6-hours before onset, AUC across the CRD (hold-out), Emory and UTHSC-MLH were, 0.90 [0.89, 0.90], 0.85 [0.85, 0.86], and 0.88 [0.86, 0.90] respectively (Table 2).

Fig 2(B) illustrates the temporal performance of the model tuned to different prediction horizons ranging from t_onset_ to 36 hrs prior. The retrospective temporal performance across Emory and UTHSC-MLH was observed to be different, with the Emory dataset suggesting a lower AUC in the hours preceding t_onset_ when compared to UTHSC-MLH which displays a better averaged performance. The aggregated performance on the UTHSC-MLH data was found to be highly variant, as illustrated with a broader 95% CI when compared to the Emory dataset.

### Model interpretability

Some features and clinical information showed more significant importance than others for prediction of ARDS. Using mean SHAP values generated from the training data, we generated a ranked list of the top 20 important features (S3 Fig). Among the top 20, the top 8 features contributed more to the generation of an alert for ARDS than the next 12. The top feature for prediction of ARDS was SpO_2_ (minimum), followed by SBP (standard deviation), younger age group (18–40), FiO_2_ (max), respiratory rate (max), O_2_ flow (max and standard deviation) and platelet count (min) in descending order.

**Fig 3:**
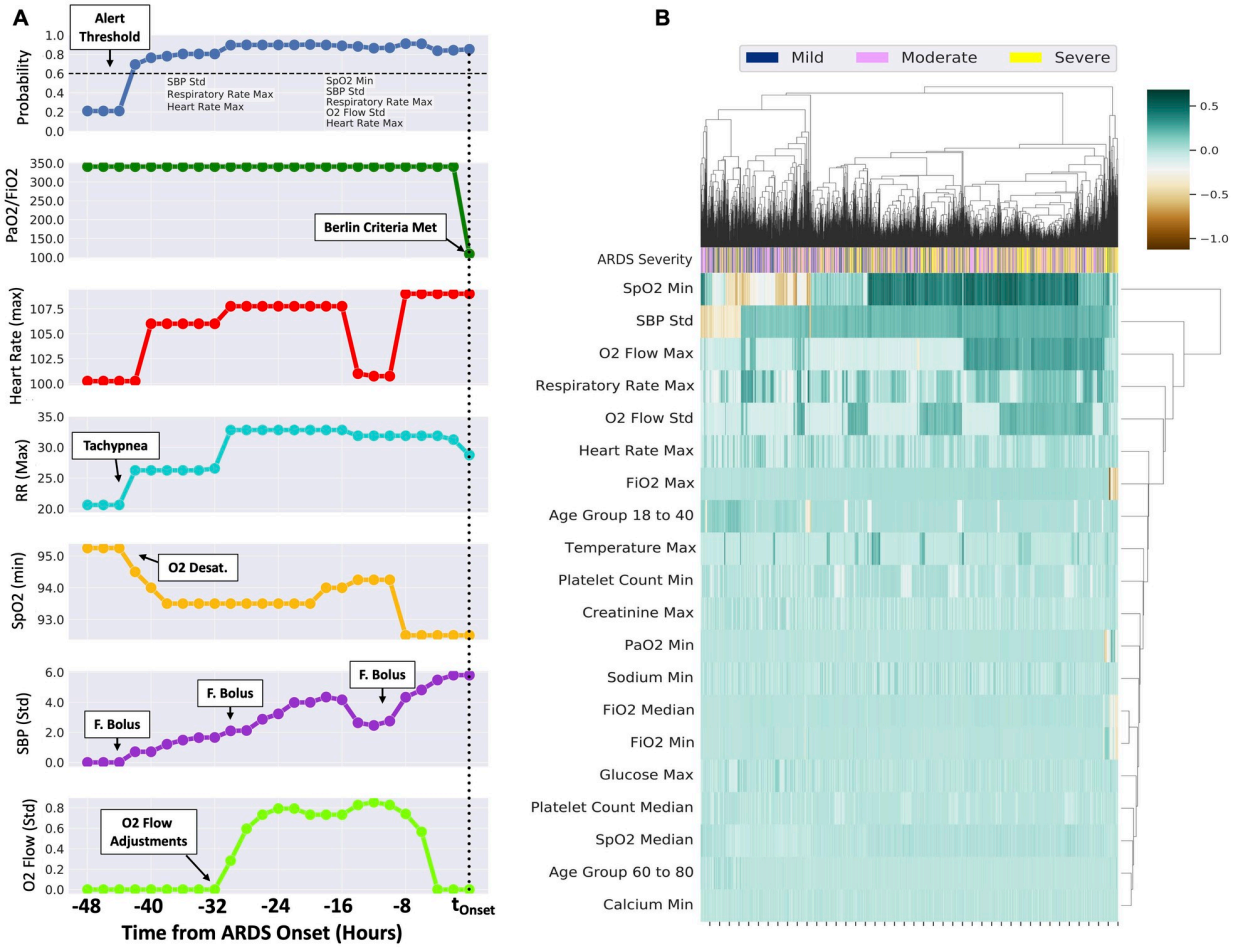
A. Example patient who develops an increased risk for ARDS over 48 hours before meeting the Berlin Criteria, the top panel illustrates probability and the second panel illustrates the PaO_2_/FiO_2_ ratio, while the third through last panels illustrate the top features identified in the model. Note that P/F ratio was not included in the model input, and illustrated for demonstration purposes. B. Illustrates the cluster heatmap of 20 most significant features derived by aggregating SHAP values for each prediction at six hours prior to t_onset_. Patients are enumerated column-wise, and clustered on disease severity, namely, mild, moderate and severe. Two groups are observed, the left consisting of the mild and moderate groups, while the right consists more of the severe (yellow) group.

Fig 3(A) illustrates an example patient who develops ARDS over a 48 hour period, SpO_2_ (minimum), SBP (standard deviation), respiratory rate (maximum), O_2_ flow (standard deviation) and heart rate (maximum) cause the probability to increase beyond the alert threshold up to 42 hours before the patient meets the severe ARDS criteria (t = 0). Resuscitative interventions were observed in the hours leading to t_Onset_. As illustrated in the figure, a series of interpretable readings, by the way of ‘important features’ are generated throughout the time period.

Fig 3(B) illustrates the clustered heatmap of the top 20 features among ARDS patients derived using the aggregated SHAP values for each prediction at six hours prior to t_Onset_. The heatmap is clustered on disease severity, namely, mild, moderate and severe. Patients are enumerated column-wise, and as illustrated in the figure, more severe patients are grouped towards the right side of the heatmap while moderate and less severe are grouped in the left. Among the less severe cluster (left side, Fig 3(B)), minimum values of the SpO_2_ and the standard deviation of SBP suppressed the probabilistic value (orange shade indicates suppression) as opposed to the severe patients group (right side, Fig 3(B)). Values of O_2_ flow (both maximum and standard deviation) contributed positively to the alert in more severe ARDS. Among the less severe cohort (left side, middle, Fig 3(B)), Age of 18–40 contributes positively to the probabilistic value, in contrast to the more severe cohort. Minimum of PaO_2_ and FiO_2_ values in the observational window were particularly important among the severe cohort. Beyond the top 10 features (SpO_2_ (*min*) till platelet count (*min*)), the remaining features add incremental value to the overall prediction.

### Cohort analysis of performance

Table 3 describes the comparisons among disease severity, which revealed statistical significance (P<0.001) among the classes, with the model performing better in severe ARDS (P/F < 100) than in mild ARDS. Table 4 describes the performance of our model when controlled against gender, age, ethnicity, and race in the CRD hold-out dataset at least 12-hours before t_Onset_. We report the average [95% CI] values of AUC, sensitivity, specificity, and PPV. Performance across age was statistically significant (P<0.001), with eARDS reporting the highest AUC of 0.93 [0.92, 0.94] for the age group of 18–40, patients over 80-years of age had the lowest AUC of 0.81 [0.81, 0.82]. Statistical significance (P<0.001) was also observed in severity, age, ethnicity and race in hold-out data of the training dataset, with the lowest performance on average among American Indian 0.85 [0.83, 0.88] and the highest performance among ‘Unknowns’ with an average AUC of 0.92 [0.91, 0.92]. A moderate statistical significance (P<0.05) was observed in sex, with females on average having an AUC greater than males.

**Table 3:** Analysis of model performance on the hold-out dataset against ARDS severity.

ARDS Severity					
Severity*	AUC	Sensitivity	Specificity	PPV	P-Value
Mild	0.83 [0.83, 0.84]	0.75 [0.74, 0.76]	0.78 [0.78, 0.79]	0.11 [0.11, 0.11]	p<0.001
Moderate	0.87 [0.87, 0.88]	0.79 [0.79, 0.80]	0.79 [0.78, 0.79]	0.27 [0.27, 0.27]	
Severe	0.91* [0.90, 0.91]	0.86 [0.85, 0.87]	0.79 [0.79, 0.80]	0.29 [0.28, 0.29]	

*statistical significance (P<0.05) by one-way ANOVA row-wise.

**Table 4:** Analysis of model performance on the hold-out CRD dataset across demographics.

	AUC	Sensitivity	Specificity	PPV	P-Value
**Gender**					
Male	0.89 [0.89, 0.89]	0.85 [0.84, 0.85]	0.76 [0.76, 0.77]	0.46 [0.45, 0.46]	p>0.05
Female	0.89 [0.89, 0.89]	0.77 [0.76, 0.78]	0.84 [0.84, 0.84]	0.49 [0.48, 0.50]	
**Age Groups** *					
18–40	0.93 [0.92, 0.94]	0.81 [0.79, 0.82]	0.93 [0.93, 0.93]	0.51 [0.50, 0.52]	p<0.001
40–60	0.89 [0.89, 0.90]	0.82 [0.81, 0.82]	0.81 [0.81, 0.82]	0.48 [0.47, 0.49]	
60–80	0.89 [0.89, 0.89]	0.81 [0.80, 0.81]	0.78 [0.78, 0.79]	0.48 [0.48, 0.49]	
80+	0.81 [0.81, 0.82]	0.82 [0.81, 0.83]	0.68 [0.68, 0.69]	0.42 [0.42, 0.43]	
**Ethnicity** *					
Not Hispanic or Latino	0.88 [0.88, 0.88]	0.80 [0.80, 0.81]	0.79 [0.78, 0.79]	0.46 [0.45, 0.46]	p<0.001
Ethnic group unknown	0.91 [0.90, 0.91]	0.87 [0.86 0.88]	0.79 [0.78, 0.80]	0.47 [0.46, 0.48]	
Hispanic or Latino	0.91 [0.91 0.91]	0.81 [0.80, 0.82]	0.87 [0.86, 0.87]	0.55 [0.54, 0.56]	
**Race** *					
Black or African American	0.91 [0.90, 0.91]	0.81 [0.80, 0.82]	0.83 [0.82, 0.83]	0.52 [0.51, 0.52]	p<0.001
White	0.87 [0.86, 0.87]	0.80 [0.79, 0.81]	0.78 [0.77, 0.78]	0.44 [0.43, 0.44]	
American Indian or Alaska Native	0.85 [0.83, 0.88]	0.71 [0.66, 0.75]	0.82 [0.79, 0.84]	0.57 [0.52, 0.63]	
Asian or Pacific islander	0.86 [0.84, 0.88]	0.86 [0.84, 0.87]	0.74 [0.73, 0.75]	0.32 [0.31, 0.34]	
Unknown Race	0.92 [0.91, 0.92]	0.88 [0.87, 0.88]	0.82 [0.81, 0.82]	0.50 [0.48, 0.52]	

*statistical significance (P<0.05) by one-way ANOVA row-wise.

### Comparison to the lung injury prediction score

We compared the performance of the eARDS model to that of the Lung Injury Prediction Score (LIPS) for predicting ARDS (Table 5). The eARDS model performed better than LIPS for predicting ARDS in COVID-19 patients, with eARDS demonstrating higher AUC across all three datasets. The eARDS model also demonstrated higher AUC than LIPS for predicting ARDS in all patients, with the exception of the Emory dataset in which the AUC was comparable.

**Table 5:** Analysis of the clinical LIPS benchmark across the three datasets.

**Prediction using LIPS (COVID-19 positives)**						
Dataset	AUC	Sensitivity	Specificity	PPV	LIPS(ARDS)	LIPS(Non-ARDS)
CRW	0.80	0.61	0.84	0.53	2.94 [2.85, 3.04]	0.95 [0.92, 0.99]
Emory	0.79	0.53	0.56	0.15	3.74 [3.39, 4.1]	1.75 [1.64, 1.87]
MLH	0.63	0.12	0.96	0.64	0.88 [0.42, 1.33]	0.42 [0.16, 0.67]
**Prediction using LIPS (All-cause)**						
Dataset	AUC	Sensitivity	Specificity	PPV	LIPS(ARDS)	LIPS(Non-ARDS)
CRW	0.80	0.63	0.83	0.38	3.00 [2.93, 3.07]	1.01 [0.99, 1.03]
Emory	0.81	0.73	0.81	0.36	3.74 [3.39, 4.09]	1.75 [1.64, 1.87]
MLH	0.61	0.10	0.96	0.60	0.88 [0.42, 1.33]	0.42 [0.16, 0.67]

## Discussion

In this study, we derived and validated a supervised machine learning model called *eARDS* for predicting the onset of ARDS in critically ill COVID-19 patients up to 36 hours before meeting the clinical criteria. In our validation, the eARDS model performed well in predicting ARDS in critically ill COVID-19 patients with an optimal prediction horizon of 12 hours before the onset of ARDS according to the Berlin definition. The high AUC and other performance characteristics of the model demonstrate the utility of the eARDS model in identifying a subset of critically ill COVID-19 patients who were at increased risk of developing ARDS. Common errors, such as missingness and incorrect data points were frequently observed among the laboratory values. These are consistent with findings from the literature [30].

The results of our study have important clinical implications, particularly from the performance of our machine learning model in early prediction of ARDS. The PPV of 0.59 and 0.48 for Emory and UTHSC-MLH validation cohorts, respectively, indicate that 48–59% of patients who were predicted to have ARDS by our model did, in fact, develop ARDS at a later time. Considering that the incidence of ARDS was 13.4% in the overall study population and 17.0% in the COVID-19 population, the PPV of 0.48–0.59 represents a significantly higher incidence of ARDS than baseline in those who were predicted by our model to develop ARDS. Our model also showed better performance in predicting severe ARDS with AUC of 0.91 compared to mild ARDS with AUC of 0.83. These characteristics may allow clinicians to promptly identify a subset of patients who are at high risk of developing ARDS, especially the severe forms of ARDS that would likely require mechanical ventilation and other advanced treatments. This early risk-stratification can inform decisions regarding various interventions, such as the timing of intubation for critically ill COVID-19 patients. While early intubation was not associated with differences in clinical outcomes or mortality in COVID-19 in one single center study, it does appear to correlate with the severity of illness and the rate of progression of disease [31, 32] [references]. Our machine learning model can predict ARDS development well before the actual disease onset, thereby alerting the clinicians of high-risk patients who may soon develop ARDS and prompting an earlier assessment of the need for intubation. In addition, early identification of high-risk patients could allow timely implementation of evidence-based treatments and strategies to prevent further lung injury. Such treatment strategies include low-tidal volume and lung protective ventilation strategies in those already receiving mechanical ventilation [33, 34], conservative fluid management and early utilization of diuretics to optimize fluid balance [35, 36]. Early prediction with our model can also prompt early corticosteroid treatment for those with severe COVID-19 who will require oxygen support or mechanical ventilation, which could mitigate the development of ARDS and improve outcomes [37–39]. Our prediction model can allow additional time for preemptive implementation of these proven strategies to attenuate lung injury in patients with progressively worsening hypoxia.

Prediction of ARDS can also improve compliance with proven ARDS management strategies such as lung-protective ventilation and prone positioning once a diagnosis is made. In a large international cohort of patients with ARDS, the diagnosis of ARDS was missed entirely in 40% of patients, with ARDS recognition ranging from only 51% in mild ARDS to 79% in severe ARDS [40]. The poor rate of recognition may be related to the complexity of the Berlin definition that utilizes both structured data and unstructured elements, such as interpretation of chest imaging that are not always definitive [18, 41]. Clinician recognition of ARDS was associated with the use of higher PEEP levels and with greater use of prone positioning, neuromuscular blockade and extracorporeal membrane oxygenation. It has also been reported that patients receiving higher tidal volumes shortly after ARDS onset have a higher mortality [42]. These findings highlight the importance of early prediction and recognition of ARDS in improving the management of ARDS, and the same principles apply to COVID-19 induced ARDS as well. Our present machine learning model and similar models can help clinicians with prediction, early recognition, and prognostication of patients at high risk of developing severe respiratory failure and ARDS. This, in turn, would allow more time for implementing strategies to avoid further lung injury, improve adherence to evidence-based therapies, and guide clinical decisions regarding treatments for severe COVID-19 and ARDS.

The example patient in Fig 3A illustrates the utility of the model in predicting ARDS onset earlier, through analysis and integration of various hemodynamic and oxygenation support variables. The initial prediction for ARDS was driven primarily by a slew of vital sign abnormalities, including the very first signs of tachycardia, tachypnea, hypoxia, and fluctuations in blood pressure. Our model was able to promptly capture the changes in these variables that suggested hemodynamic instability, and generated the initial prediction for ARDS. This alert generation was well in advance of the O_2_ flow rate adjustment [10], which can be a surrogate for the clinician’s recognition of a deteriorating patient with worsening respiratory failure. Subsequently, the standard deviation of the O_2_ flow rate sustained the ARDS prediction probability above the threshold, until the clinical criteria for ARDS was finally met. As demonstrated by this example, our model was able to integrate the earliest signs of clinical deterioration and successfully predict an increased risk of ARDS, predating the clinical suspicion or the actual onset of ARDS by several hours. Therefore, understanding the clinical context through such surrogate information may be a means by which the model recognizes worsening severity of illness.

Prior to the utilization of machine learning in predictive modeling for ARDS, a widely used clinical prediction tool for ARDS was the Lung Injury Prediction Score (LIPS) [43]. However, many of the variables used in LIPS required manual chart abstraction, and the model did not perform well when applied to settings that were different from the original validation study [44]. Machine learning models such as eARDS can automate the analysis of relevant clinical variables, and expedite the prediction of ARDS at an earlier time point than would be feasible with traditional predictive modeling. Furthermore, machine learning models can fit the data more precisely than the traditional models and result in more accurate predictions of ARDS. We demonstrate that eARDS utilized machine learning techniques to successfully analyze a complex combination of structured clinical variables that can be automatically abstracted from the EHR. Consequently, our eARDS model showed better performance for early prediction of ARDS in COVID-19 patients compared to LIPS.

The feature importance heatmaps from our model provide an indication for the most important clinical features for predicting ARDS onset in our model. Not surprisingly, features that are directly related to the patients’ respiratory status, including the minimum SpO_2_, respiratory rate, and O_2_ flow rate, were ranked among the five most important features for predicting ARDS development. The standard deviation of SBP, which is also ranked among the top five most important features for predicting ARDS, may be a surrogate of hemodynamic instability and overall clinical deterioration related to impending respiratory failure. In the clustered heatmap based on disease severity, the maximum and the standard deviation of O_2_ flow rates contributed positively to the alert in more severe ARDS, again highlighting that the features associated with the patients’ respiratory status were important in predicting ARDS development. The fact that our model placed relative importance on these factors related to vital signs and noninvasive measurements adds strength to our model. Our model could predict ARDS development without heavy reliance on invasive tests or lab values (e.g. PaO_2_), which are more likely to be obtained in patients who are already demonstrating signs of clinical deterioration and/or worsening respiratory failure. This suggests that our model can utilize vital signs and other readily available measurements to predict ARDS development before the onset of overt clinical deterioration, without heavy reliance on potentially biased availability of information.

The performance of our model with regard to age, especially the younger age group of 18–40, is also noteworthy. This may have been attributed to the lower prevalence of ARDS within this cohort, with 6% of the patients 18–40 years of age meeting ARDS criteria, while 20% met criteria within the 81+ years group. Prior literature reported that younger patients are less likely to develop ARDS and tend to suffer less severe illness from COVID-19 [45, 46]. From this, one can anticipate that younger age would not be an important feature in predicting ARDS development, but the age group 18–40 was actually one of the most important features in our model. This finding may be related to the fact that this age group contributed positively to the prediction of mild ARDS in the clustered analysis by disease severity, thus indicating an association between younger age and mild severity of ARDS.

### Limitations

There are several limitations to this study. First, we identify a select number of variables from the EMR, driven by a review of existing literature. Expanded coverage of the EMR, including deriving variables from natural language processing of the unstructured data, such as clinical notes and chest imaging studies, may improve the model’s performance and specificity. Secondly, we developed our model using a popular state-of-the-art machine learning method. However, we have not demonstrated the performance across more recent deep learning methods. Incorporating such architectures may further improve our model performance. Third, we noted that there was a high degree of missingness of variables within all three datasets; this has frequently been observed when using EMR data [30], arising from the dynamic array of possible care patterns that each patient may receive. In order to address this limitation, we incorporated methods within our pipeline that indicated missingness of a variable at each opportunity, while this effort may provide the model with some context to the nature of missingness, it is still unable to discern whether the missingness was at random or not-at-random. Further studies to this effect, particularly from prospective validation may be necessary to truly discern whether particular variables are missing or were not entered into the EMR. Finally, we developed our model and validated it using only retrospective data. A prospective validation will be essential not only for identifying potential errors and improving the performance of our model, but also to be able to implement it in clinical practice.

In conclusion, we demonstrate that machine learning methods can be applied to predictions of ARDS in patients with COVID-19. We further evaluate the performance of a general ARDS prediction model in critically ill COVID-19 patients and find that our model achieves optimal and statistically significant performance in the severe ARDS group than the mild ARDS group. Further research including the addition of blood-based biomarkers [47, 48], radiographic images, unstructured notes and high-frequency bedside monitoring data streams [49–51] may further improve the performance of the model for bedside clinical decision support.

## Supporting information

S1 Fig Consort diagram of the training dataset (CRD). (EPS)

S2 Fig The proportion of missing values for each variable across the three datasets. A higher median value signifies a greater level of missingness.
(PDF)

S3 Fig Ranking of important features in the eARDS model sorted from highest to lowest importance. (PDF)

S1 Table Variables used in machine learning model. (DOCX)
