# Supplementary figures and images for: eARDS: A multi-center validation of an interpretable machine learning algorithm of early onset Acute Respiratory Distress Syndrome (ARDS) among critically ill adults with COVID-19
Source: PLoS One. 2021 Sep 24;16(9):e0257056. doi: 10.1371/journal.pone.0257056 (PMC8462682; doi:10.1371/journal.pone.0257056)

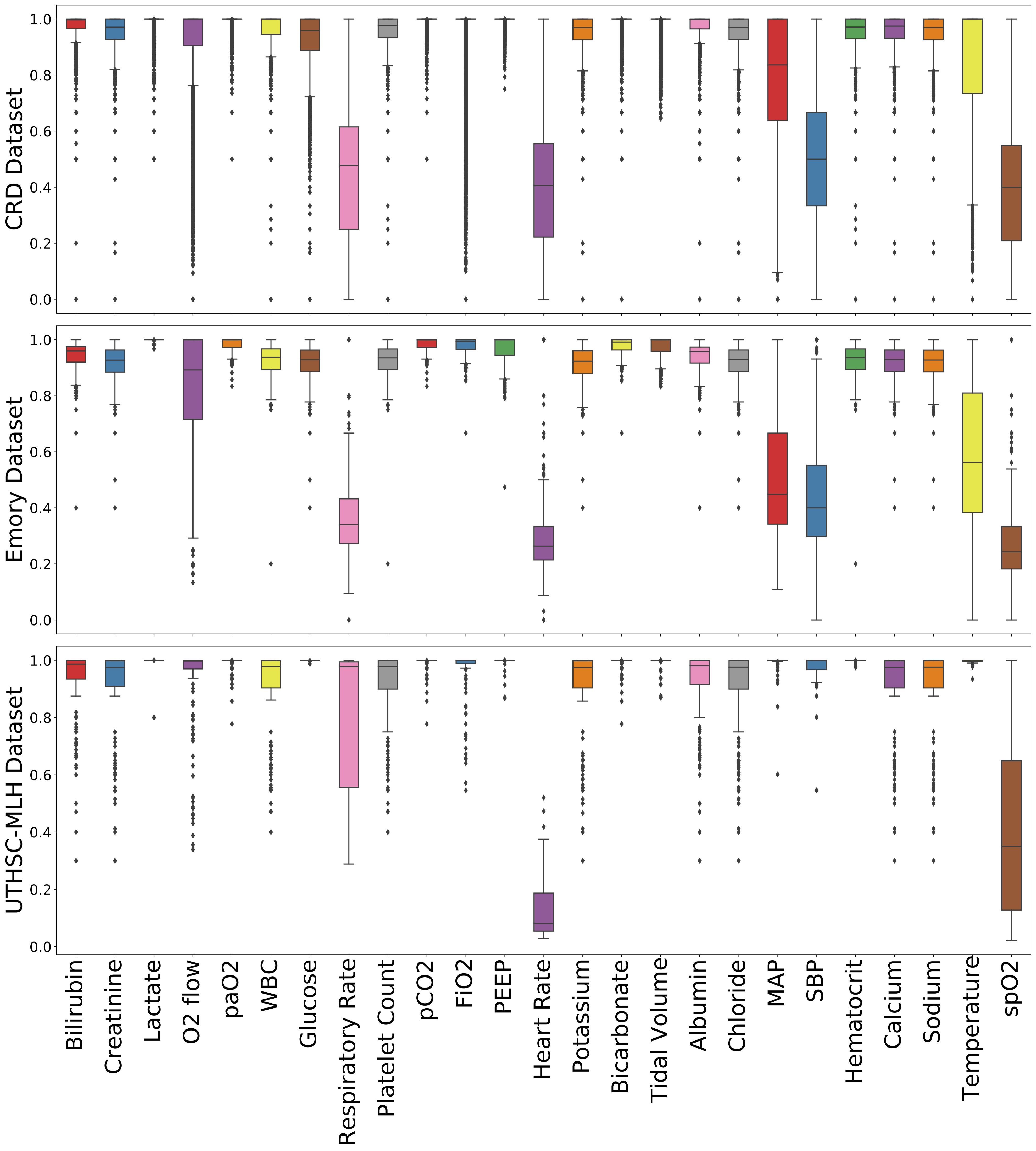

Supplement: S2 Fig — A higher median value signifies a greater level of missingness. (PDF) [file pone.0257056.s002.pdf]

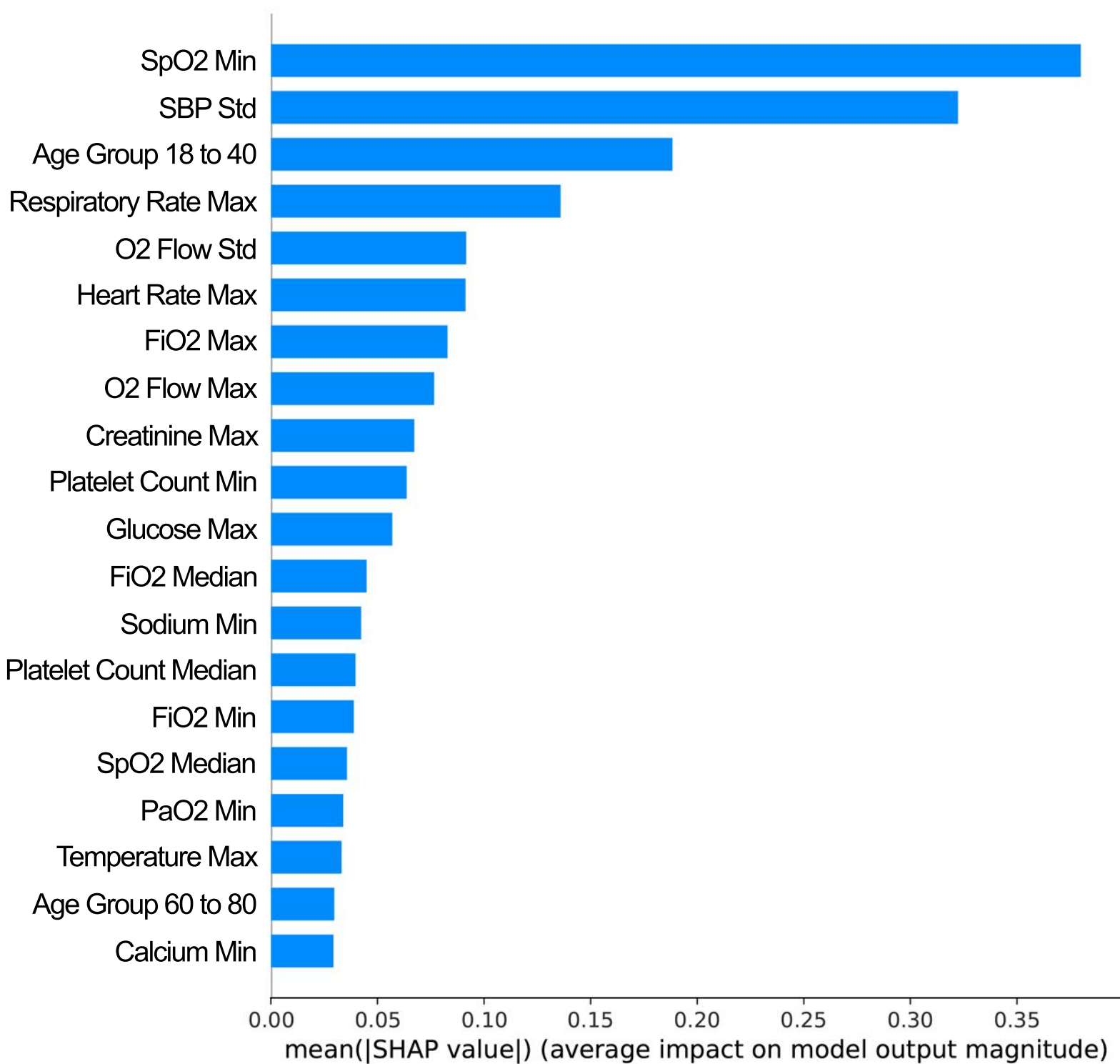

Supplement: S3 Fig — (PDF) [file pone.0257056.s003.pdf]
