# Supplementary material for: eARDS: A multi-center validation of an interpretable machine learning algorithm of early onset Acute Respiratory Distress Syndrome (ARDS) among critically ill adults with COVID-19
Source: PLoS One. 2021 Sep 24;16(9):e0257056. doi: 10.1371/journal.pone.0257056 (PMC8462682; doi:10.1371/journal.pone.0257056)
Supplement: S1 Table — (DOCX) [file pone.0257056.s004.docx]

**S1 Table. Variables used in Machine Learning Model.**

|  | **Laboratory** | **Vital** | **Demographic** | **Binary Indicator** |
| --- | --- | --- | --- | --- |
| 1. | Albumin | Heart Rate | Gender | Oxygen Therapy |
| 2. | Bicarbonate | Mean Arterial Pressure | Age group:   1. 18 to 40 2. 41 to 60 3. 61 to 80 4. 81+ | Mechanical Ventilation |
| 3. | Bilirubin | Respiratory Rate |  | Vasopressors:   1. Norepinephrine 2. Epinephrine 3. Vasopressin 4. Phenylephrine 5. Midodrine 6. Dopamine 7. Dobutamine |
| 4. | Creatinine | O_2_ flow |  | Inotropes:   1. Dobutamine 2. Milrinone |
| 5. | Glucose | Tidal Volume |  | Num. of Concurrent Vasopressors |
| 6. | Hematocrit | PEEP |  | Num. of Concurrent Inotropes |
| 7. | Platelet Count | Systolic Blood Pressure |  | Congestive Heart Failure |
| 8. | Sodium | Temperature |  | Cardiac Arrhythmias |
| 9. | WBC |  |  | Valvular Disease |
| 10. | Lactate |  |  | Hypertension |
| 11. | Chloride |  |  | Chronic Pulmonary |
| 12. | Calcium |  |  | Diabetes Uncomplicated |
| 13. | Potassium |  |  | Diabetes Complicated |
| 14. | PCO_2_ |  |  | Renal Failure |
| 15. | PaO_2_ |  |  | Liver Disease |
| 16. | FiO_2_ |  |  | Metastatic Cancer |
| 17. | SpO_2_ |  |  | Rheumatoid Arthritis |
| 18. |  |  |  | Obesity |
